# Analysis of factors affecting the prognosis of osteochondral lesions of the talus

**DOI:** 10.1007/s00264-022-05673-x

**Published:** 2022-12-27

**Authors:** Lei Bai, Yi Zhang, ShiKai Chen, Yan Bai, Jun Lu, JunKui Xu

**Affiliations:** 1 Honghui Hospital, Xi’An Jiaotong University, Xi’An, 710000 Shaanxi China; 2 Affiliated Hospital of Shaanxi University of Chinese Medicine, Xianyang, 712046 Shaanxi China; 3 First Clinical Medical College, Shaanxi University of Chinese Medicine, Xianyang, 712046 Shaanxi China; 4 Kunming Medical University, Kunming, 650000 Yunnan China; 5 Foot and Ankle Surgery Center, Honghui Hospital, Xi’An Jiaotong University, Xi’An, 710000 Shaanxi China

**Keywords:** Talus osteochondral injury, Prognosis, Related factors, Correlation

## Abstract

**Purpose:**

This study aims to analyze the correlation between the prognosis of osteochondral lesions of the talus and patient age, gender, duration of illness, and injury location, surface area, depth, and volume.

**Methods:**

A retrospective analysis of 44 patients who underwent talus osteochondral transplantation in the Department of Foot and Ankle Surgery of our hospital between January 2017 and December 2020 was performed. The clinical medical records of the patients were collected, and the location of the osteochondral lesion of the talus was determined according to the nine-division method. The surface area, depth, and volume of the osteochondral lesion of the talus were measured using mimics software in all patients. The visual analog scale (VAS), the American Orthopedic Foot and Ankle Society (AOFAS), and the SF-36 quality of life questionnaire scores were evaluated before surgery and at the last follow-up, and correlation analysis was performed.

**Results:**

Of 44 patients, 30 were followed up with a mean period of 24.33 ± 12.19 months. There were 18 men and 12 women, with an average age of 40.73 ± 10.57 years and an average disease duration of 28.30 ± 21.25 months. The VAS, AOFAS, and SF-36 scores of all patients at the last follow-up were significantly better than those before surgery. The degree of post-operative symptom improvement was not correlated with age, sex, duration of illness, and injury location, surface area, depth, and volume.

**Conclusion:**

The prognosis of osteochondral lesion of the talus is not related to patient age, gender, duration of disease, or injury location, surface area, depth, and volume.

## Introduction

Osteochondral lesion of the talus (OLT) is a common sequela of acute ankle injury. OLT includes osteochondritis dissecans (OCD), which as yet to be clearly defined. Therefore, the terms osteochondral lesion, osteochondral fracture, sheet fracture, or transtalar fracture are often used for the same lesion [1–6]. The incidence of OLT was first reported by Coltart [7] in 1951, who analyzed 25,000 fractures and found that the incidence of osteochondral injury was 0.09%. Kessler et al. [8] reported a 6.9-fold higher risk of OCD in men than in women in a multivariate logistic regression analysis of men aged 12 to 19 years. The reported incidence of OLT after ankle deformity is as high as 6.5% [9]. Most of these injuries occur after trauma (sprain or fracture) [10]. Berndt and Harty [11] experimentally demonstrated that trauma is the cause of OLT in 1951.

OLT accounts for approximately 4% of all osteochondral injuries in the human body; it usually occurs six to 12 months after the initial trauma and manifests as destruction of articular cartilage and subchondral bone [12]. Ankle sprains and fractures are considered the main causes of OLT [13]. It has been reported that 93–98% of lateral injuries and 61–70% of medial injuries are related to trauma [14, 15]. PRP has been shown to be effective in the treatment of short bone disease [16, 17]. However, its efficacy associated with the treatment of cartilage lesions in the lower limb is uncertain [18]. In patients with Hepple IV–V stage of MRI classification, talus osteochondral transplantation is the preferred treatment. Patients treated with this method achieve good post-operative results [19, 20], although the long-term efficacy remains to be determined by clinical and follow-up observations [21, 22].

The efficacy of treatments for OLT is mainly evaluated by the comparative analysis of different surgical methods. However, whether surgery is necessary and the choice of different surgical methods remain controversial issues [23]. In addition, large differences in the mid-term prognosis have been reported [24, 25]. Kim et al. analyzed prognostic factors for talar osteochondral grafts and found that patient age, sex, body mass index, duration of symptoms, location of the OLT, and the presence of subchondral cysts did not significantly affect the clinical outcomes of talar osteochondral grafts [26]. This provides a reference for further research in this field. Literature reports on the exact correlation between the prognosis of talus osteochondral injury and patient age, gender, duration of disease, and injury location, surface area, depth, and volume are lacking. Whether there is an intrinsic relationship between the prognosis of talus osteochondral injury and these factors remains to be determined, which would help the selection and optimization of clinical treatments.

## Materials and methods

### Case data

The clinical and imaging data of patients undergoing talus osteochondral transplantation at the Foot and Ankle Surgery Department of Xi’An Honghui Hospital between January 2017 and December 2020 were retrospectively reviewed. Informed consent was obtained from all patients for the operation and follow-up data collection. The study passed the ethical review and was approved by the hospital ethics committee.

Inclusion criteria were as follows: patients who failed conservative treatment for talus osteochondral injury; osteochondron grafting treatment was evaluated and approved by the patient; patients with Hepple V talus osteochondral injury determined pre-operatively; patients with a single cyst; and patients with a follow-up time of more than 12 months and complete follow-up data. Exclusion criteria were as follows: patients with ankle and knee osteoarthritis; patients with ankle deformity, infection, bone tumours, and abnormal alignment; patients with diabetic foot, rheumatoid arthritis, or gout; and patients with incompletely closed epiphysis.

### Surgical methods

**Fig. 1:**
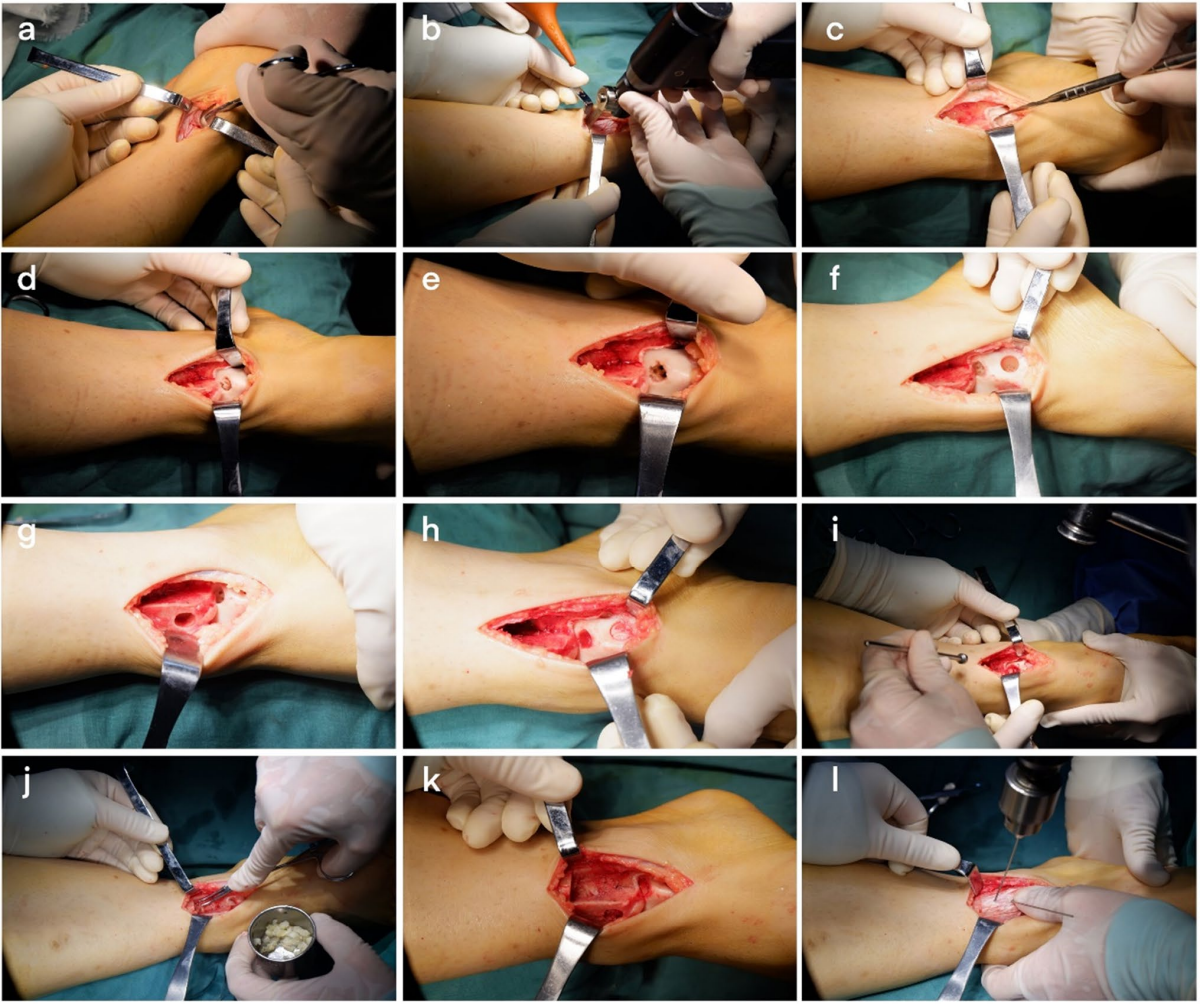
Flowchart of the surgical procedure. **a** After incision to expose the damaged area of the talus osteochondral lesion, the peeled and separated cartilage pieces can be seen locally. **b** Because the bony barrier affects the operation, a medial malleolus osteotomy was performed to fully expose the operative area. **c** After the medial malleolar osteotomy, the operative area was fully exposed, the exfoliated cartilage sheet can be seen lifted up locally, and the subchondral capsule becomes clearly visible. **d** The necrotic bone in the cystic degeneration area was cleaned and cured. **e** Microfracture was performed in the cystic degeneration area. **f** The talus from the non-weight-bearing area on the medial side of the talus was excised, with the bone column with cartilage for backup. **g** A bone column of the same size was collected from the anterior side of the distal end of the tibia. **h** The bone column was placed from the distal end of the tibia to the non-weight-bearing area on the medial side of the talus and flattened. **i** Osteotomy of the tibia was performed. An appropriate amount of cancellous bone was taken from the site and implanted into the cystic degeneration area, and the spare cartilage column was implanted and flattened. **j** Allogeneic bone granules were placed in the osteotomy site. **k** The osteotomy and tibia can be seen. The distal bone extraction sites were well filled. **l** The osteotomy block was reduced and fixed

**Fig. 2:**
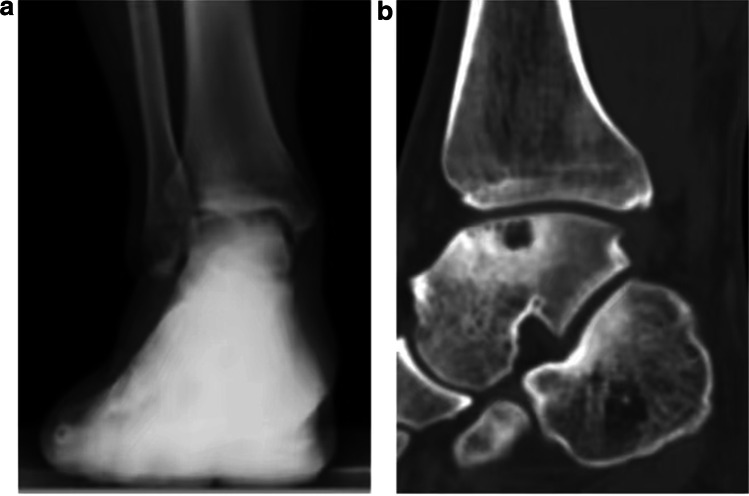
Pre-operative X-ray and CT. **a** Pre-operative X-ray showing that the local bone density of the medial talus was reduced. **b** Pre-operative CT showing that the local sac of the talus fornix becomes obvious and a cavity was formed

**Fig. 3:**
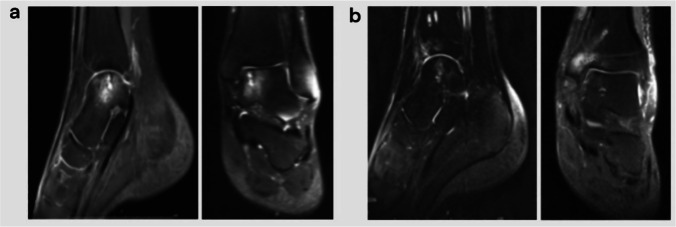
MRI before and at the last follow-up after surgery. **a** Pre-operative MRI showing cystic degeneration of the medial talus with a local high signal. **b** MRI of the patient at the last follow-up (17 months after surgery), after talus osteochondral transplantation; there is no obvious change in the medial talus. Cystic degeneration and high signal intensity were significantly reduced

The patient was admitted to the room, and after administering general anaesthesia, the lower extremity of the affected side was routinely disinfected with Aner’s iodine. A sterile surgical drape was laid, a balloon tourniquet was applied, the blood was removed, and the operation was timed. If the lesion was close to the medial side, a 5-cm arc-shaped incision was made at the anterior edge of the medial malleolus, and the skin, subcutaneous tissue, and fascia were separated layer by layer. Care should be taken to protect the saphenous vein and important nerves and extreme plantar flexion of the ankle joint. In cases of a bony obstruction at the lesion site, an oscillating saw was used to perform a tri-plane osteotomy on the medial malleolus. The medial malleolus was propped up distally to fully expose the lesioned area of the medial talus; if the lesion was close to the lateral side, the lateral malleolus was removed. An arc-shaped incision with an anterior edge of approximately 8 cm is used to separate the skin, subcutaneous tissue, and fascia layer by layer. Important blood vessels and superficial peroneal nerves were protected. The ankle was placed in extreme plantar flexion to fully expose the lesion. In cases of a bony obstruction, a pendulum saw a “Z”-shaped osteotomy was performed on the fibula. The exfoliated and necrotic cartilage of the articular surface was carefully cleaned, and the size of the lesion area was observed. A ring-shaped bone extractor with an appropriate diameter was used, and the ring-shaped bone extractor and the talus cartilage surface were maintained perpendicular to the subchondral bone. A cylindrical bone groove was drilled in the lesion area with a depth of 10–12 mm, and the cystic necrotic tissue and surrounding areas in the lesion were cleaned. For sclerotic bone, a Kirschner target was used to polish the pericystic wall until fresh blood oozed. A 3-cm-long surgical incision was made on the medial side of the talus or the medial side of the knee joint, and the cartilage surface of the medial non-weight-bearing area of the talus or the cartilage surface of the non-weight-bearing area of the medial femoral condyle was exposed. A cylindrical cartilage of appropriate length was extracted from the non-weight-bearing area with an equal diameter annular bone extractor. One or two columns were reserved, and the adjacent two columns were separated by 1 mm to avoid intersection. Then, an appropriate amount of cancellous bone was collected from the osteotomy site to implant in the cleaned cystic area, and the removed cartilage column was placed in the gap under pressure to construct the grafted bone. The cartilage surface of the column was at the same level as the cartilage surface of the talus. The ankle joint was passively moved, and the osteotomy ends of the medial malleolus and lateral malleolus were reduced and fixed with screws or plates. After satisfactory results were confirmed by intraoperative C-arm X-ray fluoroscopy, the joint cavity was washed, and the surgical incision was sutured layer by layer. Sterile gauze and bandages were used for pressure dressing to avoid post-operative complications such as joint swelling and infection. The surgical procedure is shown in Fig. 1; the pre-operative X-ray and computed tomography (CT) results are shown in Fig. 2; the pre-operative and post-operative MRI images obtained at the last three follow-up visits are shown in Fig. 3.

### Imaging data and data collection

The imaging data of all patients were obtained from the CT room of the Medical Imaging Department of our hospital (CT model: Siemens, Munich, Germany; SOMATOM Definition Flash second-generation dual-source CT; slice thickness, 1 mm). Mimics Medical version 21.0 performed three-dimensional imaging of the talus in all patients to visualize the location of talar osteochondral lesions and then measured the surface area, depth, and volume of the talar osteochondral lesions to obtain complete data.

### Description of mimics software operation steps

**Fig. 4:**
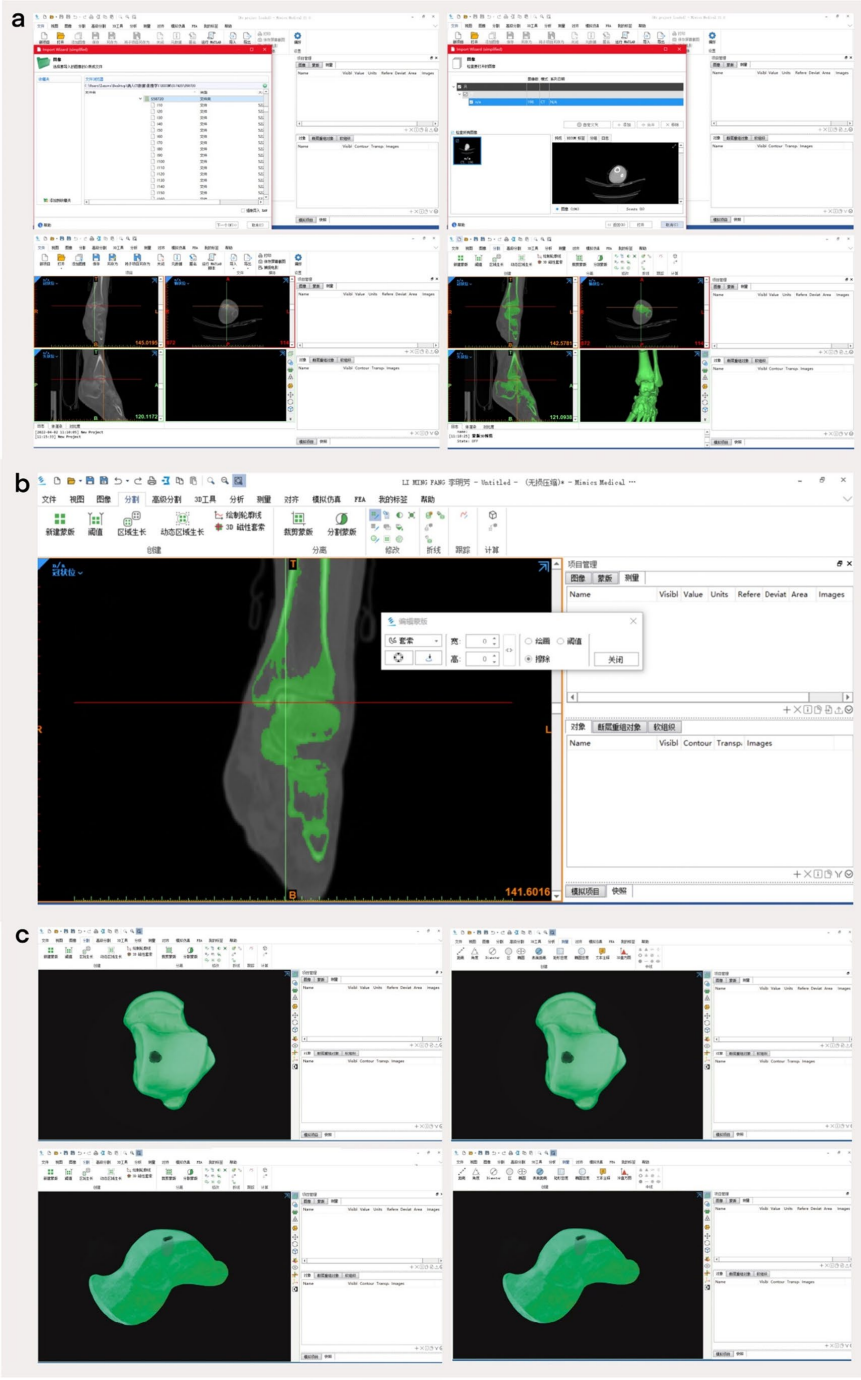
The 3D modeling procedure of the talus. **a** Import the CT data of the patient and reconstruct the 3D image of the ankle joint. **b** Extract the talus through the “Lasso” and “Erase” tools in the “Edit Mask” interface. **c** Fully display the precise location of the talus dome osteochondral injury in the top and medial views to measure the surface area and depth of the talus injury site

**Fig. 5:**
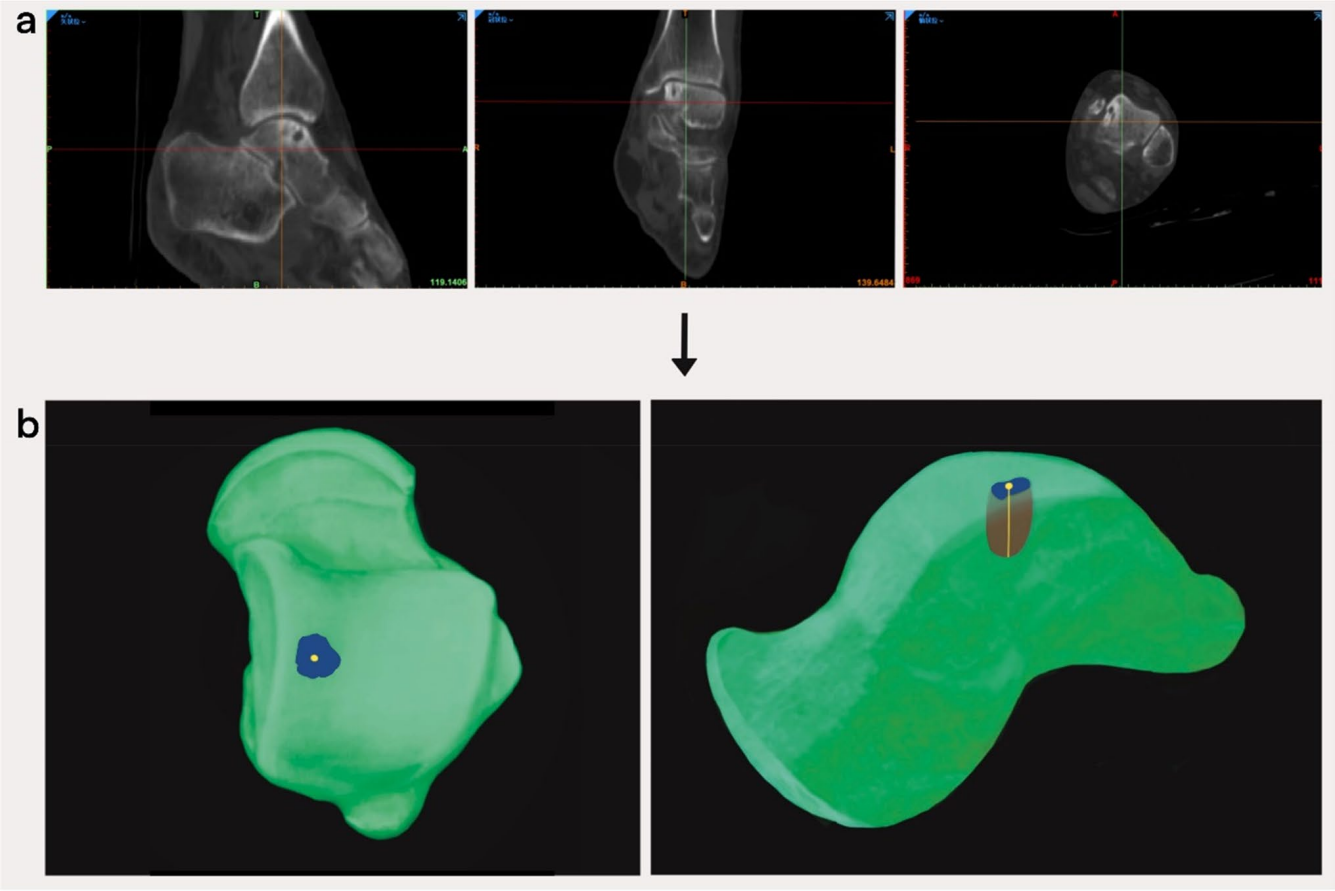
Annotation and measurement of the lesion site. **a** The location of the talus osteochondral injury was determined by CT sagittal/coronal/axial views. **b** The injury surface area (blue area) was measured in the three-dimensional view, a vertical line was drawn from the center of the injury surface area to the bottom of the cyst (yellow area) to accurately measure the depth of the injury, and the cystic area was perfused. The red area in the figure is the cystic volume

First, the complete imaging data of the patients included in the study were imported into the mimics imaging system one by one, and the images were adjusted to the appropriate size and centered in the sagittal, coronal, and axial views. The procedure used to obtain the data was as follows: select the “Segment” menu, adjust the threshold range to 117–2208 HU, check the “Fill Hole” and “Keep Maximum” options at the same time, click “OK,” and “Mask 3D Preview” buttons to obtain the ankle Full 3D skeletal image. Click the “Edit Mask” button under the “Segment” menu, select the “Lasso” and “Erase” tools, choose the sagittal/coronal/axial interface, and erase the bones other than the talus layer by layer along the joint space. After the layers have been erased, the complete 3D skeletal image of the talus can be obtained. Click the space bar to display the talus alone, and turn the mouse to fully display the precise location of the talus vault osteochondral injury (see Fig. 4). Click the “Area” and “Distance” buttons under the “Measure” menu to measure the surface area and depth of the injury site of the talus in the top view and medial view of the talus, respectively; perfuse the cystic area to measure its volume, as shown in Fig. 5.

### Partitioning method

**Fig. 6:**
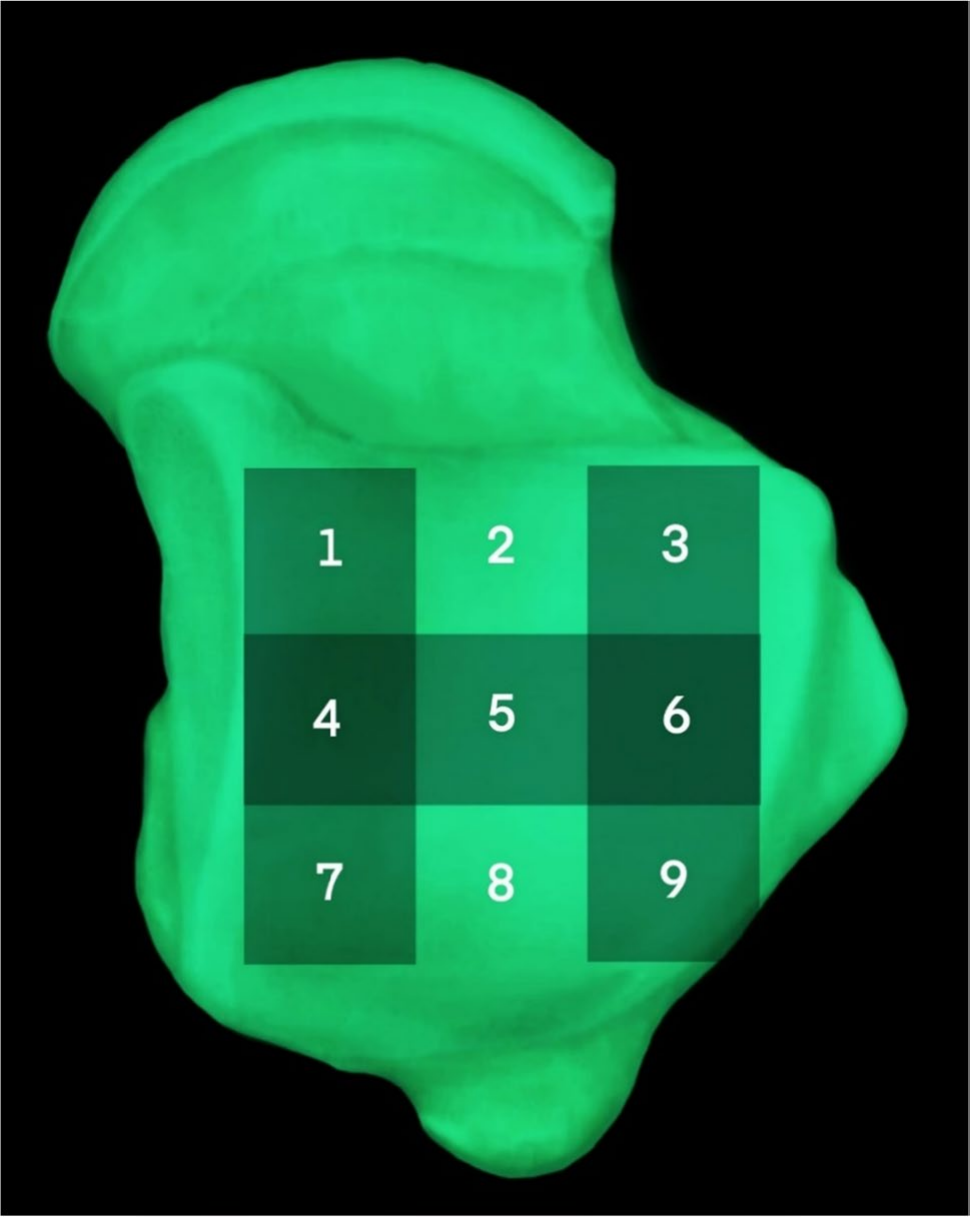
Projection labeling map of the nine-divisional surfaces of the talus dome

The lesions were localized according to the nine divisions of the talar dome surface described by Raikin et al. [27]. In the coronal plane, the articular surface of the talar vault is divided into medial, central, and lateral, and in the sagittal plane, the talus trochlea is divided into three strips, anterior, middle, and posterior. In this way, the talus surface is divided into a 3 × 3 grid comprising nine areas. The cross-regional injury was divided according to the area in which the cystic degeneration was located. If the area of injury and cystic degeneration crossed the division line, the division was based on the division of the deepest cystic degeneration. In this way, the precise location of the talar osteochondral injury in all patients in this study was determined (Fig. 6).

### Efficacy evaluation

The basic information of the patients (age, gender, average duration of illness, and average follow-up time) was collected. Before surgery and at the last follow-up, the visual analog scale (VAS) [28], the American orthopaedic foot and ankle society (AOFAS) score [29], and the SF-36 score [30] were used to evaluate the functional outcome and to perform correlation analysis of the prognosis. The VAS was used to measure pain, with 0 representing no pain and 10 representing severe pain. The AOFAS scoring scale has a total of 100 points and includes subjective and objective criteria for assessing clinical parameters. The scores are assigned as follows: 40 points for pain, 50 points for function, and 10 points for stance. The SF-36 quality of life questionnaire includes physical functioning (PF) (including 10 items), role-physical (RP) (4 items), bodily pain (BP) (2 items), general health (GH) (5 items), vitality (VT) (4 items), social functioning (SF) (2 items), role-emotional (RE) (3 items), and mental health (MH) (5 items). There are 36 items with different levels. There are four to six alternative answers for eight, according to different situations providing forward or reverse scores; a higher score indicates a greater effect of treatment. The first four dimensions belong to the physical health aspect (PCS), and the last four dimensions belong to the mental health aspect (MCS). According to the scoring method of the SF-36 scale, the initial score of the eight dimensions is calculated, and the final score = (actual initial score − theoretical minimum score)/(theoretical maximum score − theoretical minimum score) × 100.

### Statistical processing

SPSS18.0 (SPSS, Chicago, IL, USA) statistical software was used to analyze the data. The improvement of pain, function, and quality of life were expressed as the mean ± standard deviation (*x* ± *s*) using paired samples *t* tests. Analysis of variance (ANOVA) was used for the comparison of partition data. Pearson’s correlation coefficient was used for correlation analyses, and the results were expressed using the English lowercase letter *r*. Differences with a *p* value < 0.05 were considered statistically significant.

## Results

### General condition of the patients

Of 44 patients analyzed, 30 received complete follow-up, including 18 men and 12 women. The average follow-up time was 24.33 ± 12.19 months, the average age 44.20 ± 13.87 years, the average disease duration 28.30 ± 21.25 months, the average injury surface area 147.51 ± 44.01 mm^2^, the average injury depth 9.21 ± 0.73 mm, and the average injury volume 939.20 ± 217.33 mm^3^. Regarding the donor sites for osteochondral transplantation, the non-weight-bearing area of the medial femoral condyle was used in 13 cases, and the non-weight-bearing area of the talus was used in 17 cases.

### Analysis of various evaluation indicators

At the last follow-up after the operation, the VAS, AOFAS, and SF-36 scores of all patients were significantly better than those before surgery (*P* < 0.05). Among them, the VAS score decreased from 6.60 ± 0.56 before surgery to 1.83 ± 0.65 at the last follow-up, and the pain symptoms were significantly improved, whereas the AOFAS score increased from 32.77 ± 11.72 before surgery to 90.47 ± 1.96 at the last follow-up. Joint pain symptoms and activity function were greatly improved. SF-36 quality of life scores was evaluated in two dimensions, physical and psychological. Physiologically, the score increased from 110.90 ± 9.57 before surgery to 317.90 ± 9.22 after, and the health status was greatly improved; psychologically, the score increased from 139.03 ± 10.65 before surgery to 353.20 ± 10.36 after, and the psychological distress and burden caused by the disease also improved (Table 1). The VAS, AOFAS, and SF-36 scores at the last follow-up after surgery were not significantly correlated with age and duration of illness (all *P* > 0.05) (Table 2) or with gender (all *P* > 0.05) (Table 3). The VAS, AOFAS, and SF-36 scores at the last follow-up after surgery were not significantly correlated with the injury surface area, depth, and volume (all *P* > 0.05) (Table 4). Among the 30 patients who received complete follow-up, 15 cases had an injury in zone 4, ten cases in zone 6, five cases in zone 7, and zero cases in other zones. There was no significant correlation between the VAS, AOFAS, and SF-36 scores at the last follow-up after surgery and the injury zone (all *P* > 0.05), as shown in Table 5.

**Table 1:** Changes of VAS, AOFAS, and SF-36 scores before surgery and at the last follow-up

Evaluation indicators	Preoperative	Last follow-up after surgery	*t* value	*P* value
VAS	6.60 ± 0.56	1.83 ± 0.65	30.42	< 0.05
AOFAS	32.77 ± 11.72	90.47 ± 1.96	− 26.61	< 0.05
SF-36(PF)	37.60 ± 6.53	80.47 ± 5.69	− 35.80	< 0.05
SF-36(RP)	6.93 ± 4.97	45.37 ± 5.85	− 25.80	< 0.05
SF-36(BP)	33.00 ± 4.16	96.03 ± 1.92	− 72.43	< 0.05
SF-36(GH)	33.37 ± 3.85	96.03 ± 1.45	− 91.21	< 0.05
SF-36(VT)	45.73 ± 6.08	81.73 ± 5.58	− 23.07	< 0.05
SF-36(SF)	46.70 ± 4.98	96.00 ± 1.84	− 50.16	< 0.05
SF-36(RE)	5.43 ± 3.56	96.10 ± 1.75	− 143.22	< 0.05
SF-36(MH)	41.17 ± 6.70	79.37 ± 5.85	− 22.27	< 0.05
SF-36(PCS)	110.90 ± 9.57	317.90 ± 9.22	− 97.35	< 0.05
SF-36(MCS)	139.03 ± 10.65	353.20 ± 10.36	− 82.62	< 0.05

**Table 2:** Correlation of VAS, AOFAS, and SF-36 scores with age and duration of illness at the last follow-up after surgery

Evaluation indicators		*r* value	*P* value
Age	VAS	− 0.011	> 0.05
	AOFAS	0.114	> 0.05
	SF-36(PF)	0.089	> 0.05
	SF-36(RP)	− 0.133	> 0.05
	SF-36(BP)	− 0.335	> 0.05
	SF-36(GH)	0.265	> 0.05
	SF-36(VT)	0.100	> 0.05
	SF-36(SF)	− 0.072	> 0.05
	SF-36(RE)	− 0.006	> 0.05
	SF-36(MH)	0.117	> 0.05
	SF-36(PCS)	− 0.089	> 0.05
	SF-36(MCS)	0.104	> 0.05
Duration of illness	VAS	− 0.279	> 0.05
	AOFAS	0.103	> 0.05
	SF-36(PF)	− 0.212	> 0.05
	SF-36(RP)	0.020	> 0.05
	SF-36(BP)	0.222	> 0.05
	SF-36(GH)	− 0.011	> 0.05
	SF-36(VT)	0.108	> 0.05
	SF-36(SF)	0.005	> 0.05
	SF-36(RE)	0.078	> 0.05
	SF-36(MH)	− 0.115	> 0.05
	SF-36(PCS)	0.079	> 0.05
	SF-36(MCS)	0.092	> 0.05

**Table 3:** Correlation of VAS, AOFAS, and SF-36 scores with gender at the last follow-up after surgery

	Evaluation indicators	*t* value	*P* value
Gender	VAS	0.757	> 0.05
	AOFAS	1.149	> 0.05
	SF-36(PF)	0.702	> 0.05
	SF-36(RP)	1.938	> 0.05
	SF-36(BP)	0.872	> 0.05
	SF-36(GH)	0.719	> 0.05
	SF-36(VT)	1.079	> 0.05
	SF-36(SF)	0.704	> 0.05
	SF-36(RE)	1.415	> 0.05
	SF-36(MH)	1.605	> 0.05
	SF-36(PCS)	1.079	> 0.05
	SF-36(MCS)	0.727	> 0.05

**Table 4:** Correlation of VAS, AOFAS, and SF-36 scores with injury surface area, depth, and volume at the last follow-up after surgery

Evaluation indicators		*r* value	*P* value
Surface area	VAS	− 0.160	> 0.05
	AOFAS	0.115	> 0.05
	SF-36(PF)	0.029	> 0.05
	SF-36(RP)	0.257	> 0.05
	SF-36(BP)	0.258	> 0.05
	SF-36(GH)	0.208	> 0.05
	SF-36(VT)	− 0.115	> 0.05
	SF-36(SF)	0.079	> 0.05
	SF-36(RE)	0.092	> 0.05
	SF-36(MH)	0.071	> 0.05
	SF-36(PCS)	0.267	> 0.05
	SF-36(MCS)	0.008	> 0.05
Depth	VAS	− 0.146	> 0.05
	AOFAS	0.092	> 0.05
	SF-36(PF)	− 0.013	> 0.05
	SF-36(RP)	0.055	> 0.05
	SF-36(BP)	0.354	> 0.05
	SF-36(GH)	0.126	> 0.05
	SF-36(VT)	0.117	> 0.05
	SF-36(SF)	0.268	> 0.05
	SF-36(RE)	− 0.040	> 0.05
	SF-36(MH)	0.318	> 0.05
	F-36(PCS)	0.121	> 0.05
	F-36(MCS)	0.284	> 0.05
Volume	VAS	− 0.279	> 0.05
	AOFAS	0.103	> 0.05
	SF-36(PF)	− 0.212	> 0.05
	SF-36(RP)	0.020	> 0.05
	SF-36(BP)	0.222	> 0.05
	SF-36(GH)	− 0.011	> 0.05
	SF-36(VT)	− 0.108	> 0.05
	SF-36(SF)	0.005	> 0.05
	SF-36(RE)	− 0.078	> 0.05
	SF-36(MH)	0.155	> 0.05
	SF-36(PCS)	− 0.048	> 0.05
	SF-36(MCS)	− 0.151	> 0.05

**Table 5:** Correlation of VAS, AOFAS, and SF-36 scores with injury zone at the last follow-up after surgery

	Evaluation indicators	*F* value	*P* value
Damage zone	VAS	0.993	> 0.05
	AOFAS	0.251	> 0.05
	SF-36(PF)	0.634	> 0.05
	SF-36(RP)	1.178	> 0.05
	SF-36(BP)	1.918	> 0.05
	SF-36(GH)	0.972	> 0.05
	SF-36(VT)	0.729	> 0.05
	SF-36(SF)	1.179	> 0.05
	SF-36(RE)	0.707	> 0.05
	SF-36(MH)	1.655	> 0.05
	SF-36(PCS)	2.495	> 0.05
	SF-36(MCS)	2.163	> 0.05

## Discussion

The ankle is an important joint in the human body, and the tibiotalar articular surface plays an important role. OLT may be associated with up to 73% of ankle fractures and chronic ankle soft tissue injuries [31, 32]. The surface of the talus is composed of cartilage, subchondral bone, and a weak subchondral bone plate. The hyaline cartilage that covers the surface of the talus has poor elasticity and is thinner than other cartilages. It has poor deformation ability under strong external stress. Damage to the subchondral bone plate greatly increases the probability of subchondral cystic degeneration or osteonecrosis of the talus [20]. Advances in imaging technology and changes in the exercise habits of people have resulted in an increase in talus osteochondral injuries in clinical practice, and most of them are caused by poor articular surface reduction after ankle fractures, long-term high-intensity exercise, chronic strain, and drug-related injury. In patients with autometabolic and other diseases, selecting an adequate treatment strategy according to the different grades of injury generally leads to good clinical results, although the prognosis is often affected by multiple factors [33]. Among patients with larger injuries and severe clinical symptoms, those in Hepple stage IV–V often undergo autologous osteochondral transplantation [34].

In this study, the VAS, AOFAS, and SF-36 scores of all patients were significantly better than those before surgery. This suggests that patients treated with talus osteochondral transplantation can achieve better clinical results. Hangody et al. studied a large number of autologous osteochondral transplantation cases, 98 of which were distributed in the talus. According to the Hannover scoring system for evaluating the postoperative recovery of patients, up to 93% of patients had good to excellent ankle function, and only 3% of patients had residual cartilage donor site pain [35]. Paul et al. retrospectively analyzed 113 cases of talus osteochondral injury to study the effect of autologous osteochondral transplantation on resumption of sports. The results showed that although all patients were able to return to sports, exercise mode and exercise intensity were important factors affecting recovery [36]. The results of the study demonstrate the effectiveness of autologous osteochondral transplantation in the treatment of talus osteochondral injury. The biggest advantage of autologous osteochondral transplantation is that the cartilage is a fresh autologous graft and its cartilage activity is good, although post-operative pain at the donor site is a possible complication. Therefore, the surrounding cartilage should be protected as much as possible during the operation to reduce damage to the soft tissue and nerves at the donor site [37].

The results of this study showed that VAS, AOFAS, and SF-36 scores at the last follow-up after surgery were not significantly correlated with age, gender, and duration of illness. The average age of the 30 patients with complete follow-up in this study was 40.73 ± 10.57 years, and comparison of various indicators between before and after surgery showed no correlation between prognosis and age. Of the 30 patients, there were 18 men and 12 women, and gender had no effect on the prognosis. Patients with complete follow-up had an average disease duration of 28.30 ± 21.25 months; the shortest disease duration was three months and the longest 78 months. Disease duration had no effect on the prognosis. Kim et al. analyzed the prognostic factors of talar osteochondral transplantation and found that age, gender, and duration of symptoms did not significantly affect the clinical outcome of talar osteochondral transplantation [26], which is consistent with the results of this study.

The results of this study showed that VAS, AOFAS, and SF-36 scores at the last follow-up after surgery were not significantly associated with injury location. Due to the limited sample size of this study, among the cases with complete follow-up, the cystic degeneration zones were mainly distributed in zones 4, 6, and 7, and the number of cases in other zones was zero. Whether the scores are correlated with other injury zones remains unclear. Kim et al. reported the prognostic factors of talar osteochondral grafts and found that the location of the OLT and the presence of subchondral cysts did not significantly affect the clinical outcomes of talar osteochondral grafts [38], which is consistent with this study. The impact of injury location on the long-term prognosis of osteochondral transplantation has not been studied in detail to date. It has been reported in the literature that autologous osteochondral transplantation is used to treat OLT. The long-term follow-up case data need to be further supported [39].

In this study, the VAS, AOFAS, and SF-36 scores at the last postoperative follow-up were not significantly associated with injury surface area and depth. Haleem et al. reported that OLT size was not a significant predictor of outcome in osteochondral grafts, and the use of multiple grafts did not adversely affect outcomes [40]. Gautier et al. reported that the size of the OLT surface area ranges from 70 to 420 mm^2^, and there is no evidence to support that the size of the defect affects the degree of pain or disease progression [41]. This conclusion is consistent with our findings. Regarding the correlation between the depth of injury and the prognosis of OLT, there are few studies addressing this issue. Haleem et al. reported that the size of OLT cysts does not affect the treatment outcome [40]. In this study, the average injury depth was 9.31 ± 2.72 mm, with a range of 6.59–12.03 mm. All patients achieved satisfactory results after autologous osteochondral transplantation, and injury depth did not affect treatment outcomes. Taken together, the results indicate that there is no clinical reference to predict the prognosis based on the surface area and depth of injury.

In this study, the VAS, AOFAS, and SF-36 scores at the last follow-up after surgery were not significantly correlated with the lesion volume. The present study is the first to report this result. Among all the patients included in this study, the smallest injury volume was 578 mm^3^, the largest 1405 mm^3^, and the average injury volume 939.20 ± 217.33 mm^3^. All patients obtained satisfactory results with talus osteochondral transplantation. All patients improved post-operatively, suggesting that there is no significant correlation between the injury volume and prognosis. The reason for this conclusion could be as follows: during the operation, we performed cancellous bone grafting on the cystic area to ensure that a gap was not present after osteochondral transplantation; therefore, the cystic area was fully filled and improved. The autologous cancellous bone has better activity, which can accelerate the healing of the transplanted area. Therefore, regardless of the size of the cyst, as long as the cystic area is adequately filled and supported, patients can achieve good post-operative outcomes.

The results of this study show that the prognosis of talus osteochondral injury is not significantly correlated with age, gender, duration of disease, and the location, surface area, depth, and volume of the injury. These results may help the selection and optimization of the treatment plan for talus osteochondral injuries. Further studies should be performed with a larger sample size and longer-term follow-up.

## Conclusion

For patients with talus osteochondral injury, talus osteochondral transplantation can achieve good clinical results, and the prognosis should be fully affirmed. The findings suggest that age, sex, duration of illness, and location, surface area, depth, and volume of lesions do not affect the clinical outcomes of osteochondral transplantation.

## Explanation

Of the 44 original data collected in this study, a total of 30 patients met the inclusion and exclusion criteria and received full follow-ups, with a mean age of 40.73 ± 10.57 years. By analyzing the raw data, we found that patients who were too young and too old did not meet the inclusion criteria. The inclusion criteria were as follows: (1) patients who did not respond to conservative treatment of talus osteochondral injury; (2) osteochondron grafting treatment was evaluated and approved by the patient; (3) patients with pre-operative assessment of Hepple V. talus cartilage injury; (4) patients with single cystic mutations; and (5) patients with post-operative follow-up time greater than 12 months and complete follow-up data). Most of the patients in this study were 30–50 years of age, and the minimum and maximum values were outliers and were excluded, so the confounding bias caused by age factors was reduced. Due to the lack of current references in this area, there was no high-quality literature evidence to support a direct correlation between age and the prognosis of talus osteochondral injury. The results of this study showed that the prognosis of talus cartilage injury was independent of the age of disease. We are now working to continue to expand the sample size and conduct further research to verify this conclusion.

## Data Availability

Yes.
